# Fluctuations in local shear-fault energy produce unique and dominating strengthening in metastable complex concentrated alloys

**DOI:** 10.1073/pnas.2209188120

**Published:** 2023-03-13

**Authors:** Wei Li, Shuang Lyu, Yue Chen, Alfonso H. W. Ngan

**Affiliations:** ^a^Department of Mechanical Engineering, Faculty of Engineering, The University of Hong Kong, Hong Kong 999077, China; ^b^Shenzhen Institute of Research and Innovation, The University of Hong Kong, Shenzhen 518057, China; ^c^Department of Engineering Mechanics, College of Aerospace Engineering, Chongqing University, Chongqing 400044, China

**Keywords:** shear-fault fluctuations, dislocation resistance, special atomic motifs, modeling of strengthening, high-entropy alloys

## Abstract

Local chemical short-range ordering (SRO) and spatial fluctuations of planar fault energy are important features of multi-element and metastable complex concentrated alloys (CCAs). Arising from them, dislocations in such alloys are distinctively wavy in both static and migrating conditions; yet, such effects on strength have remained unknown. In this work, molecular dynamics simulations are used to show that the wavy configurations of dislocations and their jumpy motion in a prototypic CCA of NiCoCr are due to the local fluctuations of the energy of SRO shear-faulting that accompanies dislocation motion, with the dislocation getting pinned at sites of hard atomic motifs (HAMs) associated with high local shear-fault energies. Unlike the global averaged shear-fault energy which in general will subdue on successive dislocation passes, the local fluctuations in the fault energy always remain in a CCA, thus offering a strength contribution that is unique in such alloys. Analysis of the magnitude of this form of dislocation resistance shows that this is dominating over contributions due to elastic misfit of alloying elements and is in good agreement with strengths predicted from molecular dynamics simulations and experiments. This work has unfolded the physical basis of strength in CCAs, which is important for the development of these alloys into useful structural materials.

## Significance

Complex concentrated alloys (CCAs) with multi elements in equiatomic ratios are exotic alloys but what governs their strength is currently not clearly understood. This work has proven the prime origin of strength in CCAs as arising from the fluctuations (not the averaged value) of the shear-fault energy, which is an important feature of CCAs unrecognized beforehand. Through identification of the local hard atomic motifs far beyond the traditional concept of the globally averaged short-range ordering parameters, this work has unfolded the physical basis of strength in CCAs and provides important clues for designing high-strength CCAs by focusing on the alloy composition to produce large fault-energy fluctuations.

High-entropy alloys (HEAs) were originally defined as alloys composed of five or more alloying elements with concentrations at 5 to 35% (1) according to the assumption that a high configurational entropy of mixing could stabilize the formation of single-phase solid solutions. However, mixing entropy has significant effects on the Gibbs free energy of an alloy system only at elevated temperatures, and practical alloys are usually homogenized through thermal processing such as annealing or quenching before being used at ambient temperatures. The configurational entropy is also known to distinctly decrease with lowering of annealing temperature (2, 3) rather than preserving the ideal random mixing as at high temperatures. Specifically, complex concentrated alloys (CCAs) exhibit local chemical ordering (2, 4, 5), phase decomposition (6, 7), and fluctuations of staking fault energy (2, 8) after given thermal processing due to the intrinsic metastable nature in CCAs (9).

Numerous efforts have been made to understand how the complex microstructures mentioned above give rise to the physical basis of strength in HEAs and CCAs (10–12). Destruction of SRO by dislocation motion has been stipulated as an important contribution to strength in these alloys (13), while current strengthening models are based primarily on classical solid-solution strengthening due to the elastic modulus and size misfit of alloying elements (11, 12). Although SRO effects have been considered through taking the energy change of the system as a strengthening contribution when specific chemical bonds are broken (4), the bond-breaking contribution is actually on the basis of mean binding energy and globally averaged SRO parameters, with local fluctuations ignored. In fact, a distinctive feature of HEAs and CCAs is that their dislocations are highly wavy when at rest or traversing, as shown in previous simulations (2, 4, 14, 15) and experiments (16, 17). Yet, a comprehensive understanding of such wavy configurations of dislocations and their jumpy motion, and what forms the key basis of strength in CCAs, is still lacking.

Here, we study the above issues by molecular dynamics (MD) simulations on a representative metastable NiCoCr CCA, which is a face-centered cubic (FCC) single-phase solid solution with excellent mechanical properties, especially better than those of the CrMnFeCoNi Cantor alloy (18–21). SRO in NiCoCr has been confirmed by experiments and atomistic simulations: Local Co-Cr ordering pairs were identified from molecular dynamics (MD) simulations (2) and density-functional theory (DFT) calculations (8), as well as experiments using energy-filtered transmission electron microscopy (5) and extended X-ray absorption fine structure spectroscopy techniques (22). In order to understand the underlying strengthening mechanism in the NiCoCr CCA, here in this work, MD simulations were carried out on species of this alloy with different SRO states generated by Monte Carlo MD at different annealing temperatures. The pinning sites of the dislocations and SRO effects on their waviness and jumpy glide were studied. By accounting for the line tension effects of the wavy dislocation configurations, a form of dislocation resistance unique to CCAs is identified and analyzed.

## 1. Jumpy Glide, Pinning, and Waviness of Dislocations in NiCoCr

**Fig. 1.**
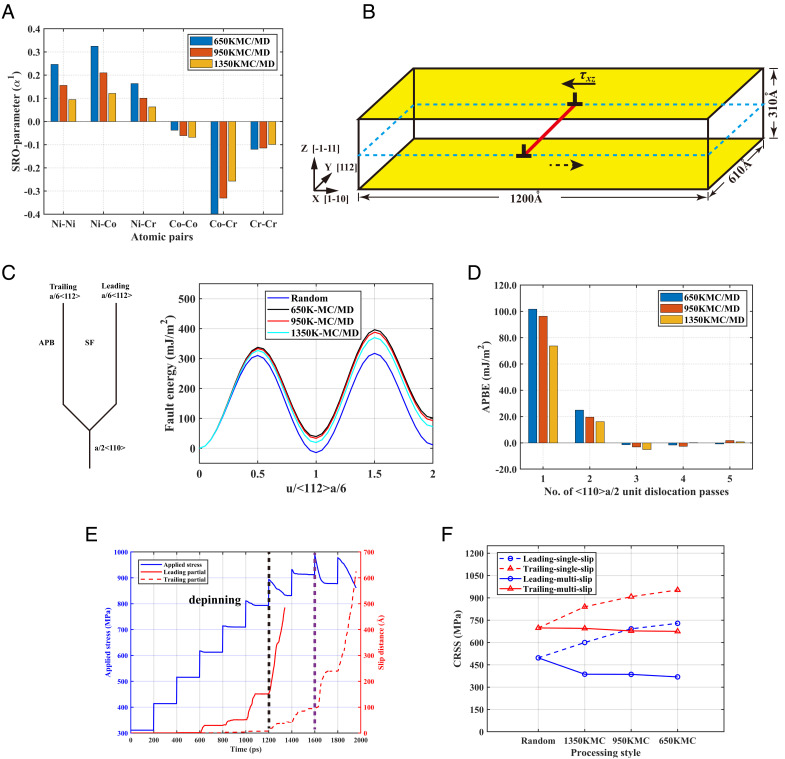
MC/MD simulations of the NiCoCr alloy of different SRO states. (*A*) SRO parameters for different atomic pairs in the MC-relaxed samples produced at various annealing temperatures. (*B*) Schematic of simulation cell for ½<110> edge dislocation motion; external stress is applied on two thin yellow slabs with thickness of 1.2 nm at the lower and upper Z surfaces. (*C*) MD-computed fault-energy landscapes associated with the passage of the leading and trailing 1/6<112> partial dislocations. (*D*) APBE for multiple passes of ½<110> unit dislocations. (*E*) Applied stress and average positions of the leading and trailing partials obtained from MD simulations at 5 K for the alloy annealed at 950 K, with stress applied at intervals of 100 MPa. More data given in *SI Appendix*, Fig. S2. (*F*) MD-simulated CRSS of leading and trailing Shockley partials at various annealing states for single and five passes (details in *SI Appendix*, Fig. S3) of dislocations, respectively.

The MD simulations carried out are summarized in Fig. 1. Here, hybrid Monte Carlo/molecular dynamics (MC/MD) was employed to obtain different alloy species for mimicking different annealing conditions, comprising a random state where the three elements of Ni, Co, and Cr in equal ratio are randomly distributed in the simulation cell, and three other states relaxed from the random state at 1,350 K, 950 K, and 650 K (details in Materials and Methods). The calculated Warren–Cowley parameters characterizing the degree of SRO (23) for different annealed states is given in Fig. 1*A*, which are comparable to previous results (2). For dislocation simulations, the simulation cells used comprised 20.3 million atoms with the three orthogonal directions as X-$\left[1,\bar{1},0\right]$ , Y-$\left[112\right]$ , and Z-$\left[\;,\bar{1},\bar{1},1\right]$ as shown in Fig. 1*B*, with periodic boundary conditions (PBCs) employed along X and Y, while free boundary conditions were used on Z surfaces for stress application.

As the NiCoCr alloy has the FCC crystal structure, its unit ½<110> dislocation is dissociated into two 1/6<112> Shockley partial dislocations separated by a stacking fault with energy SFE or $\gamma_{\mathrm{SF}}$ , as shown in Fig. 1*C*. While the FCC crystal structure is recovered by the advancement of the trailing 1/6<112> Shockley partial, for CCAs with SRO, certain chemical disorder will remain in the wake of the trailing partial in comparison with the original SRO ahead of the leading partial; the residual chemical disorder is thus manifested in a trailing antiphase boundary (APB) with specific energy APBE or $\gamma_{\mathrm{APB}}$ . The MD-calculated energy landscapes at 0 K for the random and the SRO samples are given in Fig. 1*C*. The first valleys, corresponding to the passage of the leading Shockley partial, correspond to the intrinsic SFEs (24) and complex stacking fault energies (CSFEs) for random and SRO samples, respectively. On decreasing annealing temperature from 1,350 K to 650 K, i.e., increasing the SRO degree, the SFE of −14.4 mJm^−2^ for the random state increases to 19.2, 33.4, and 39.2 mJm^−2^. After the trailing partial passes through the cell, a nonzero APBE remains in all states as in ordered alloys, and as the annealing temperature decreases or SRO increases, the APBE increases from 11.5 mJm^−2^ to 72.5, 92.7, and 101.2 mJm^−2^. However, successive shears of the ½<110> dislocation system will gradually destroy the SRO and hence the APB, as indicated by the fall of the APBE on successive dislocation passes in Fig. 1*D*.

In previous simulations of dislocations in FCC HEAs (4, 15), the entire dissociated ½<110> system comprising the Shockley partials and the stacking fault glides as a whole on the {111} slip plane. However, in the present NiCoCr alloy with negative or weakly positive SFE, the two Shockley partials are pinned and move rather independently when the applied stress is below the critical resolved shear stress (CRSS) of the full dislocation, resulting in a spectrum of metastable dissociation distances as analyzed by Vaid et al. (25). Fig. 1*E* shows the glide distances of the two partials at different applied stresses at 5 K for the sample annealed at 950 K. In this annealed state and others, both partials show jerky movements under a smaller applied stress, and as the applied stress increases, the leading partial is first unpinned and continuously glides through the periodic image of the simulation cell (the slip distance of the leading partial in the periodic image is not plotted in this figure and in *SI Appendix*, Figs. S2 and S3) before the trailing partial starts to move. This implies that the stable separation between partials could be spanning nearly half of the simulation cell due to the low or negative SFE. As the applied stress increases up to the CRSS of the full dislocation, the entire extended dislocation begins to move. The CRSSs of the two partials at 5 K in different annealed states are given in Fig. 1*F*, indicating that the CRSS of the trailing partial is larger than that of the leading partial. On the first pass of the ½<110> dislocation system, the predicted CRSSs of both partial dislocations show significant strengthening attributed to the increase in SRO with decreasing of the annealing temperature, in consistence with the trends of the CSFEs and APBEs in Fig. 1*D* and previous simulations (2). However, after a few passes of the ½<110> dislocation system, the strengthening due to the SRO gained at lower annealing temperatures diminishes, as shown in Fig. 1*F*. In particular, there is a softening of ~100 MPa, compared with the random state, for the leading partial.

**Fig. 2.**
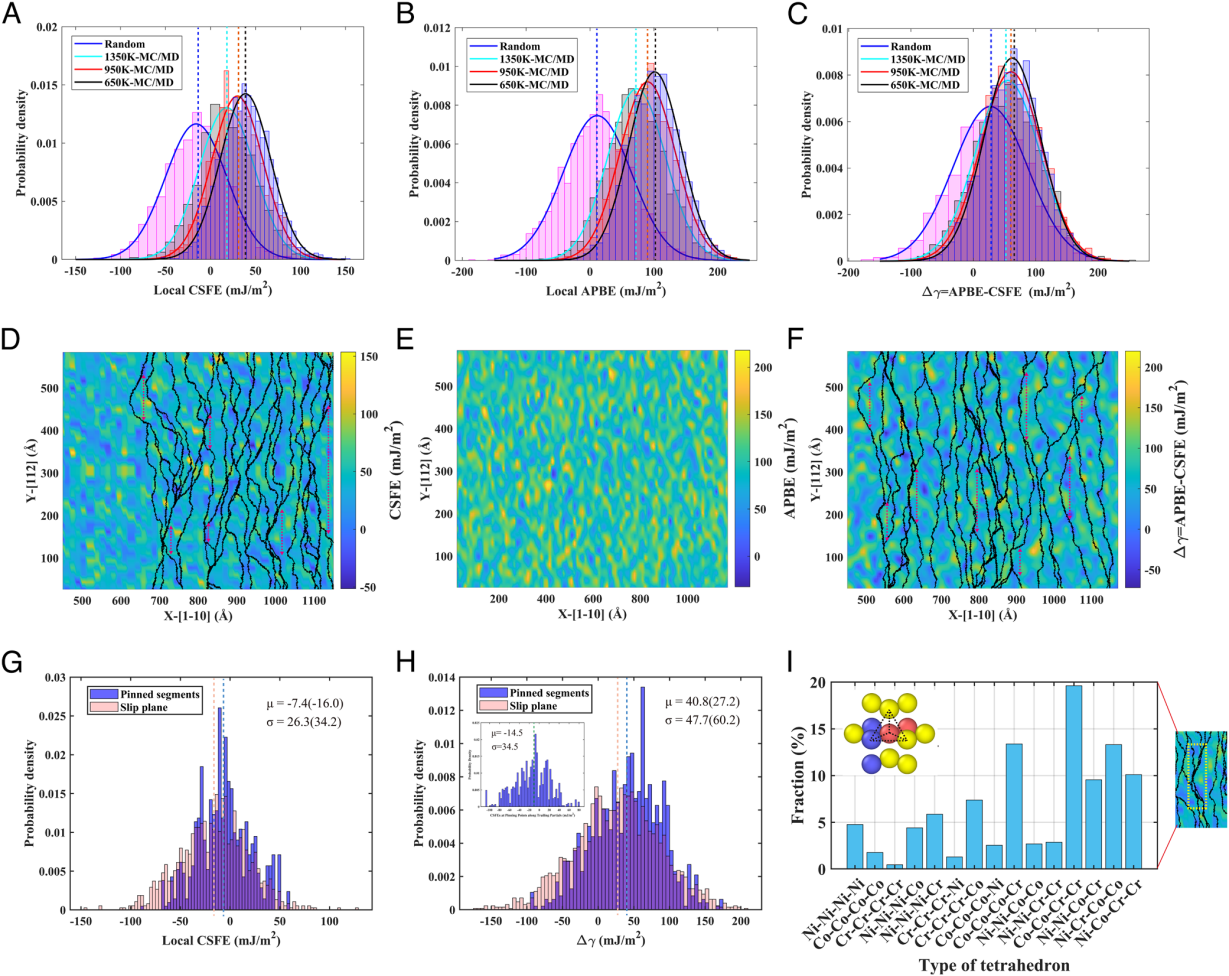
Dislocation waviness and pinning sites. (*A*–*C*) Statistical distributions of locally harvested CSFE (*A*) and APBE (*B*) and the difference Δγ = APBE−CSFE (*C*) on the {111} slip plane for different annealed states of the alloy. Dashed lines denote mean values. (*D* and *F*) Successive snapshots of leading (*D*) and trailing (*F*) Shockley partials as they traverse from left to right on the {111} slip plane in time intervals of 6 ps and 14 ps, respectively, superimposed on local CSFE (*D*) and $\Delta_{\gamma }$ (*E*) maps over the slip-plane domain for the annealed state at 650 K. Double-arrowed lines denote pinned dislocation segments. (*E*) Local APBE map over the slip plane. (*G* and *H*) Statistical distribution of local CSFE (*G*) and Δγ (*H*) for the random alloy state harvested around the pinned dislocation segments for the leading (*G*) and trailing (*H*) partials, respectively (μ and σ are mean and SD in mJm^−2^, respectively; values in parentheses are for the whole slip plane). More data given in *SI Appendix*, Fig. S4. (*I*) “HAMs” (hard atomic motifs) of the pinning sites that correspond to high gamma; tetrahedron distribution on the slip planes given in *SI Appendix* Fig. S5.

The CSFEs and APBEs shown in Fig. 1*C* are global values averaged over the slip plane, and they serve to illustrate the contribution of ordering to strengthening. However, the planar fault energies of CCAs are locally varying (2, 8, 16) over the slip plane due to the spatial variations of the alloying elements and chemical ordering. Fig. 2 *A*–*C* show the statistical distributions of the CSFE and APBE and $\Delta_{\gamma }=APBE-CSFE$ for the different annealed states studied, respectively. With increasing SRO degree, the mean CSFE and APBE increase in consistence with the results in Fig. 1*C*, and correspondingly, the mean $\Delta_{\gamma }$ also increases with the SRO degree while its fluctuations decrease. The local values of the CSFE and APBE and $\Delta_{\gamma }$ , respectively, on the {111} plane for the example of the alloy state annealed at 650 K are given in Fig. 2 *D*–*F*, where the cross-sectional area used for local fault-energy calculations is 15 × 17 ${Å}^{2}$ . Successive snapshots of the leading and trailing partials in time intervals of 6 ps and 14 ps, respectively, are superimposed on the local fault-energy maps, as shown in Fig. 2 *D* and *F*, as they traverse from the left of the simulation cell to the right. The motion of the partials is evidently jerky, which corresponds well to the jumpy flow in Fig. 1*E*, and between the jumps, the dislocations are pinned at locations with high values of the corresponding local fault energy (examples marked by double-arrowed lines). Fig. 2 *G* and *H* show the statistical distribution of the local fault energies harvested along pinned dislocation segments for the random alloy state, for which the variations of the local fault energies are the largest among the different alloy states. It can be seen that the local fault energies along the pinned dislocation segments are significantly higher than the global averaged values in this case. For instance, the mean CSFE along the pinned leading Shockley segments in Fig. 2*G* is −7.4 mJm^−2^ versus the average value of −16 mJm^−2^ for the alloy from Fig. 2*A*. The atomic arrangements at selected pinning sites of the Shockley dislocations are also analyzed, and the results, shown in Fig. 2*I*, indicate that the pinning points that correspond to high local fault energies are predominantly Co-Co-Cr-Cr, Co-Co-Co-Cr and Ni-Cr-Co-Co tetrahedrons, which we call “hard atomic motifs” (HAMs) here.

Therefore, to summarize, the present MD simulations indicate that dislocation motion in the NiCoCr CCA is jerky, with the dislocations adopting highly wavy configurations due to pinning at sites with high local fault energies. This aspect will be analyzed in detail in the next section.

## 2. Dislocation Resistance due to Fault-Energy Fluctuations

### 2.1. Mean-Field Mobility of Dislocations.

**Fig. 3.**
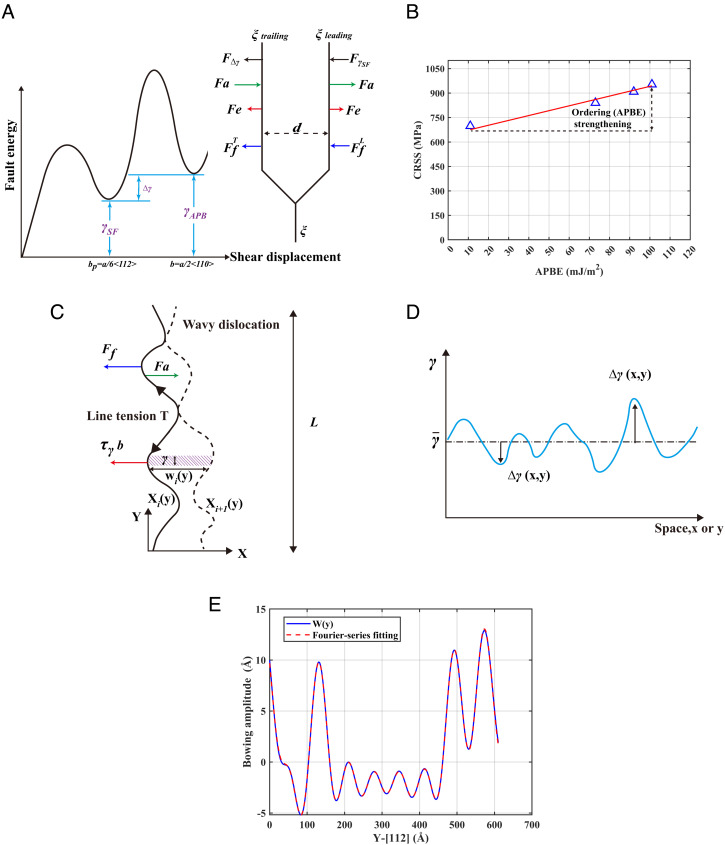
Theoretical analysis of dislocation resistance. (*A*) Forces acting on 1/6<112> partial dislocations and fault energies: $F_{f}^{L}$ and $F_{f}^{T}$ are lattice friction on leading and trailing partials, respectively, $F_{e}$ is mutual elastic interaction, $F_{a}=\tau_{a}b$ is force due to applied stress, $F_{\gamma SF}$ and $F_{\Delta \gamma }$ are forces due to $\gamma_{\mathrm{SF}}$ and $\gamma_{\mathrm{APB}}$ . $\Delta \gamma =\gamma_{\mathrm{APB}}-\gamma_{\mathrm{SF}}$ is the difference in fault energy on either side of the trailing 1/6<112> Shockley. (*B*) MD-calculated CRSS for trailing partial versus APBE for different annealing states. (*C*) Two successive snapshots of a jumping dislocation, with $w\left(y\right)$ being the distance swept in between, *F_a_* the applied force, *F_f_* the lattice friction, and $\tau_{\gamma }$ the resistance stress due to the stacking fault. (*D*) Schematic representation of the fault-energy field $\gamma \left(x,y\right)$ . (*E*) Example of MD-computed $w\left(y\right)$ and fitting by the Fourier series.

Fig. 3*A* shows the various Peach–Koehler forces (forces on unit length of dislocation) acting on the two 1/6<112> Shockley partial dislocations in the present alloy system with SRO contributions. Let us first consider the case of mean-field mobility, i.e., the average mobility of a long-enough dislocation with local fluctuations along the dislocation length ignored. The motion equations are given as follows:

[1] $$mv_{L}=\tau_{a}b+F_{e}-\gamma_{\mathrm{SF}}-F_{f}^{L},$$

[2] $$mv_{T}=\tau_{a}b-F_{e}-\Delta \gamma -F_{f}^{T},$$

where $b=a/\sqrt{6}$ , m and v are the inverse of mobility and velocity of the dislocation, and all quantities refer to the averages along the leading (*L*) or trailing (*T*) partial. When $v_{L,T}>0$ , $F_{f}^{L,T}$ reaches a critical value $F_{f}^{c}$ which is the kinematic lattice friction. When $v_{L,T}=0$ , i.e., the dislocation is not moving, the lattice friction $F_{f}^{L,T}$ can be any value between $\pm F_{f}^{c}$ to balance the other forces acting on the dislocation. Two cases are imminent:

Case I—when the leading partial just starts to move and the trailing partial is still stuck. Then, $F_{f}^{L}=F_{f}^{c}$ , $F_{f}^{T}<F_{f}^{c}$ , $v_{L,T}=0$ . Adding up Eqs. **1** and **2**,

[3] $$\tau_{a}={\tau_{a}}^{L}=\frac{\gamma_{\mathrm{APB}}}{2b}+\frac{\left(F_{f}^{c}+F_{f}^{T}\right)}{2b},$$

which is the CRSS to move the leading partial.

Case II—when the trailing partial starts to move, and the leading partial is already moving. Then, $F_{f}^{L,T}=F_{f}^{c}$ , $v_{T}=0$ , $v_{L}>0$ , and

[4] $$\tau_{a}={\tau_{a}}^{T}=\frac{\gamma_{\mathrm{APB}}}{2b}+\frac{F_{f}^{c}}{b}+mv_{L}\approx \frac{\gamma_{\mathrm{APB}}}{2b}+\frac{F_{f}^{c}}{b}>{\tau_{a}}^{L}.$$

In Eq. **3**, the friction $F_{f}^{T}$ is a changeable quantity called to balance the forces acting on the trailing partial before it begins to move, and as such, it is dependent on the initial separation of the two partials. However, ${\tau_{a}}^{T}$ from Eq. **4** is a well-defined value as $\gamma_{\mathrm{APB}}$ and $F_{f}^{c}$ are constants for a given alloy. Since $\left|F_{f}^{T}\right|<F_{f}^{c}$ , from Eq. **3**, the lower and upper bounds for ${\tau_{a}}^{L}$ are as follows:

[5] $$\frac{\gamma_{\mathrm{APB}}}{2b}<{\tau_{a}}^{L}<{\tau_{a}}^{T}.$$

We first assume $F_{f}^{c}$ as a constant for the random and annealed samples. The MD-calculated CRSSs and average APBEs from Fig. 1 *C* and *F* are fitted to Eq. **4** as shown in Fig. 3*B*. Here, the slope of the fitting line corresponds to 1/(2*b*) from Eq. **4**, with $b=a/\sqrt{6}$ for the Shockley partial, where a= 3.557 Å is the lattice constant from the present MD simulation which is also comparable to the experimental value of 3.575 Å (26). The intercept of the fitting equation is 675 MPa, which, for a mean-field straight dislocation as described by Eq. **4**, would be the Peierls stress $F_{f}^{c}/b$ of the trailing partial in the random state of the alloy. However, it is important to note that for the CRSS data predicted by MD in Fig. 3*B*, the dislocations are highly wavy as seen in Fig. 2, and so, instead of the mean-field Peierls stress, a significant contribution to the 675 MPa actually arises from the line tension of the wavy dislocations, as discussed in the next section. In any case, the linear increasing trend of the MD-predicted CRSS with the APBE in Fig. 3*B* is in good agreement with Eq. **4**, and this confirms that the mean-field dislocation mobility is hampered by APBE strengthening due to the SRO in the annealed states compared to the random state. However, as can be seen from Fig. 3*B*, the amount of APBE strengthening is only about 300 MPa for the state with the highest SRO (the 650 K annealed state) compared to the much higher basic strength of ~680 MPa for the random state. Moreover, as shown in Fig. 1*D*, the APBE subdues quickly on successful dislocation passes, and so, the APBE strengthening demonstrated here is actually unimportant for macroscopic slip. The key understanding of strength in CCAs would still require an understanding of the basic strength of the random alloy state, which is pursued below.

### 2.2. Mobility of Wavy Dislocations.

As indicated by the results in Fig. 2, dislocations are highly wavy in the present alloy due to interactions with sites of high local fault energies and special HAMs. Furthermore, as can be seen from the *Insets* of Fig. 2 *A* and *C*, the random state of the alloy has the largest fluctuations in the local fault energy. Here, the contribution to the CRSS based on the local fault-energy fluctuations is deduced.

Fig. 3*C* shows the jump displacement $w\left(y\right)$ of a wavy dislocation between two dwelling states and the various forces acting. Balancing the work done as the dislocation undergoes the jump gives

[6] $$\begin{array}{c} \tau_{\gamma }b\int_{0}^{L}\;w\;\left(y\right)dy=\frac{T}{2}\int_{0}^{L}\;{\left[w^{\prime }\left(y\right)\right]}^{2}dy+\int_{0}^{L}\;\left(\int_{0}^{w(y)}\gamma \left(x,y\right)dx\right)dy. \end{array}$$

Here, the first term is the work done by the net force $\left(F_{a}-F_{f}\right)$ which is equal to $\tau_{\gamma }b$ , where $\tau_{\gamma }$ is the resistance due to the stacking fault; the second term is the change in self-energy (line tension) of the dislocation due to its change in line length during the jump, and the third term is the work done against the fault energy which is in general a field $\gamma \left(x,y\right)$ over the slip plane. Line tension T may be written as $T=\alpha \mu b^{2}$ , where μ is shear modulus, and α depends on the dislocation character inner and outer cutoff radii of the dislocation.

The dislocation waviness is due to the fluctuating $\gamma \left(x,y\right)$ field where local regions of high γ pin the dislocation (resulting in zero or small w ), as further advancement of the dislocation would produce faulting with a high energy (Fig. 2). Therefore, the line-tension term is also a consequence of the nonuniform $\gamma \left(x,y\right)$ field, so that both terms on the right side of Eq. **6** are due to $\gamma \left(x,y\right)$ . The $\gamma \left(x,y\right)$ field is spatially fluctuating as schematically shown in Fig. 3*D*, and can be written as follows:

[7] $$\gamma \left(x,\;y\right)=\bar{\gamma }+\Delta \gamma \left(x,\;y\right),$$

where $\bar{\gamma }$ is the average fault energy over the slip plane (hence it is no longer a field of *x* and *y*), and $\Delta \gamma \left(x,y\right)$ is the fluctuating component of $\gamma \left(x,y\right)$ that averages to zero. The fault-induced resistance $\tau_{\gamma }$ above may therefore be considered to be composed of two corresponding parts: $\tau_{\gamma }=\tau_{\overset{-}{\gamma }}+\tau_{\Delta \gamma }$ , where, from Eq. **6**,

[8] $$\tau_{\bar{\gamma }}b=\bar{\gamma },$$

is the old term in Eq. **3** for a “mean-field” dislocation, and $\tau_{\Delta \gamma }$ is given by

[9] $$\begin{array}{c} \tau_{\Delta \gamma }b\int_{0}^{L}w\left(y\right)dy=\frac{T}{2}\int_{0}^{L}{\left[w^{\prime }\left(y\right)\right]}^{2}dy\\ \;+\int_{0}^{L}\left(\int_{0}^{w(y)}\Delta \gamma \left(x,\;y\right)dx\right)dy. \end{array}$$

As the $\bar{\gamma }$ diminishes on successive dislocation passes as shown in Fig. 1*D*, $\tau_{\overset{-}{\gamma }}$ from Eq. **8** would also disappear gradually on successive dislocation passes as shown in Fig. 1*F*, and so, this term is unimportant for macroscopic slip. However, the fluctuation $\Delta \gamma \left(x,y\right)$ should always remain and so should be the $\tau_{\Delta \gamma }$ from Eq. **9**. The fluctuating $\Delta \gamma \left(x,y\right)$ is a unique feature of HEAs and CCAs, and so, the $\tau_{\Delta \gamma }$ is also a unique resistance for CCAs.

Of the two terms on the right side of Eq. **9**, we note that $\int_{0}^{L}\left(\int_{0}^{w(y)}\Delta \gamma \left(x,\;y\right)dx\right)dy/\int_{0}^{L}w\left(y\right)dy$ is the average value of Δγ(x,y) in the slipped region, which, for a long-enough dislocation, should also approach zero. Hence, Eq. **9** becomes

[10] $$\tau_{\Delta \gamma }\approx \frac{T}{2b}\frac{\int_{0}^{L}{\left[w^{\prime }\left(y\right)\right]}^{2}dy}{\int_{0}^{L}w\left(y\right)dy}.$$

Note that although $\int_{0}^{L}\left(\int_{0}^{w(y)}\Delta \gamma \left(x,\;y\right)dx\right)dy$ averages to zero for a long-enough dislocation, the line tension term in Eq. **10** is still due to the fluctuating displacement $w\left(y\right)$ of the dislocation and hence the fluctuating $\Delta \gamma \left(x,y\right)$ field.

A general and versatile representation of the jump displacement $w\left(y\right)$ is a Fourier series of the form:

[11] $$\begin{array}{l} w\left(y\right)=\lambda_{0}+\sum_{i=1}^{N}\left(a_{i}\sin\frac{2\text{π}y}{l_{i}}+b_{i}\cos\frac{2\text{π}y}{l_{i}}\right)\\ \;\;\;\;\;\;\;\;\;=\lambda_{0}+\sum_{i=1}^{N}\lambda_{i}\sin\left(\frac{2\text{π}y}{l_{i}}+\phi_{i}\right), \end{array}$$

where $\lambda_{i}=\sqrt{{a_{i}}^{2}+{b_{i}}^{2}}$ (Fig. 3*E* shows an example of accurate fitting to MD-computed dislocation shape). Substituting Eq. **11** into Eq. **10**, as the domain length L→∞,

[12] $$\begin{array}{c} \begin{array}{c} \int_{0}^{L}{\left[w^{\prime }\left(y\right)\right]}^{2}dy=\int_{0}^{L}{\left[\sum_{i\geq 1}2\text{π}k_{i}\cos\left(\frac{2\text{π}y}{l_{i}}+\phi_{i}\right)\right]}^{2}dy\\ ={\left(2\text{π}\right)}^{2}\sum_{i\geq 1}\left(k_{i}{}^{2}\right)\int_{0}^{L}\cos^{2}\left(\frac{2\text{π}y}{l_{i}}+\phi_{i}\right)dy+{\left(2\text{π}\right)}^{2}\\ \;\;\;\;\;\sum_{i\neq j}k_{i}k_{j}\int_{0}^{L}\cos\left(\frac{2\text{π}y}{l_{i}}+\phi_{i}\right)\cos\left(\frac{2\text{π}y}{l_{j}}+\phi_{j}\right)dy\\ \approx 2\pi^{2}L\sum_{i\geq 1}k_{i}{}^{2}. \end{array} \end{array}$$

Here, $\int_{0}^{L}\cos^{2}\left(\frac{2\pi y}{\ell_{i}}+\phi_{i}\right)dy=\frac{L}{2}+O\left(1\right)$, $\begin{array}{c} \int_{0}^{L}\cos\left(\frac{2\pi y}{\ell_{i}}+\phi_{i}\right) \end{array}$ $\begin{array}{c} \cos\left(\frac{2\pi y}{\ell_{j}}+\phi_{j}\right)dy=O\left(1\right) \end{array}$ if i≠j . Hence as L→∞ , only the factor *L*/2 prevails in the first summation in the second step of Eq. **12**, which leads to the third step. Similarly,

[13] $$\begin{array}{c} \int_{0}^{L}w\left(y\right)dy=\lambda_{0}L+\sum_{i\geq 1}\lambda_{i}\int_{0}^{L}\sin\left(\frac{2\text{π}y}{l_{i}}+\phi_{i}\right)dy\approx \lambda_{0}L. \end{array}$$

Therefore, Eq. **10** reduces to

[14] $$\tau_{\Delta \gamma }\approx \frac{\alpha \mu b\pi^{2}\sum_{i\geq 1}{k_{i}}^{2}}{\lambda_{0}},$$

where $k_{i}=\lambda_{i}/\ell_{i}$ ( i≥1 ) is the aspect ratio, with $\lambda_{i}$ and $\ell_{i}$ being the bowing amplitude and length of the $i^{\mathrm{th}}$ harmonics of the dislocation jump displacement.

## 3. Analysis of Alloy Strength

As mentioned in section 2, the partial dislocations glide separately rather than as a whole initially when the required stresses for their movement reached. As the applied stress increases, the leading partial continuously glides before the trailing partial starts to move, therefore, for the leading partial, a repulsive force arising from the unslipped image trailing partial gradually increases. Correspondingly, a pulling force arising from the gliding image leading partial acts on the unslipped trailing partial. This is actually an artificial strengthening arising from the periodic image dislocations due to the periodic boundary conditions applied. Besides, the plasticity in the present NiCoCr alloy is accommodated through the glide of partial dislocations, the CRSS of which is $\tau_{\mathrm{xz}}\cos\left(30^{\circ }\right)$ , where $\tau_{\mathrm{xz}}$ is the applied stress in the <110> direction on {111} planes. Therefore, to allow comparison with experiments, all $\tau_{\mathrm{MD}}$ and CRSSs from the simulations should be corrected as,

[15] $${\tau_{\mathrm{MD}}=\tau }_{\mathrm{xz}}\cos\left(30^{\circ }\right)\pm \tau_{\mathrm{image}},$$

where “+” applies to trailing partials and “−” to leading partials. Here, $\tau_{\mathrm{image}}=\frac{\mathrm{Gb}}{2\pi (1-v)x}$ , where *x* (~550 Å) is the distance between the gliding leading partial and the unslipped image trailing partial, shear modulus *G* = 59 GPa, Poisson’s ratio ν=0.3 , and thus, $\tau_{\mathrm{image}}\approx 61MPa.$

Next, we consider the lattice friction or Peierls stress. Zhang et al. (27) incorporated lattice distortion effects into the classical Peierls–Nabarro model for the lattice friction by scaling the interplanar potential (or generalized stacking fault energy) by a factor that is stochastically distributed according to the normal distribution with mean 1 and SD Δ. Their model predicts the Peierls stress to be

[16] $$\tau_{p}=\tau_{p}^{0}\times \langle \tilde{\tau_{p}}\rangle ,$$

where $\tau_{p}^{0}=\frac{2G}{1-\nu }\exp\left[-\frac{2\mathrm{\pi w}}{b}\right]$ is the classical Peierls stress for FCC crystals, and $\langle \tilde{\tau_{p}}\rangle$ is a factor that increases from 1 to 1.71 for Δ increasing from 0 to 0.45. For the present alloy, the $\tau_{p}^{0}$ for a Shockley partial is calculated to be ~58 MPa [ G = 59 GPa, ν=0.3 , and $\frac{\omega }{b}=1.27$ (26)], and even with a large $\langle \tilde{\tau_{p}}\rangle$ of 1.71 applied, the Peierls stress $\tau_{p}$ is estimated to be ~98 MPa, which is only a small contribution to the basic strength of ~680 MPa in Fig. 3*B*.

**Fig. 4.**
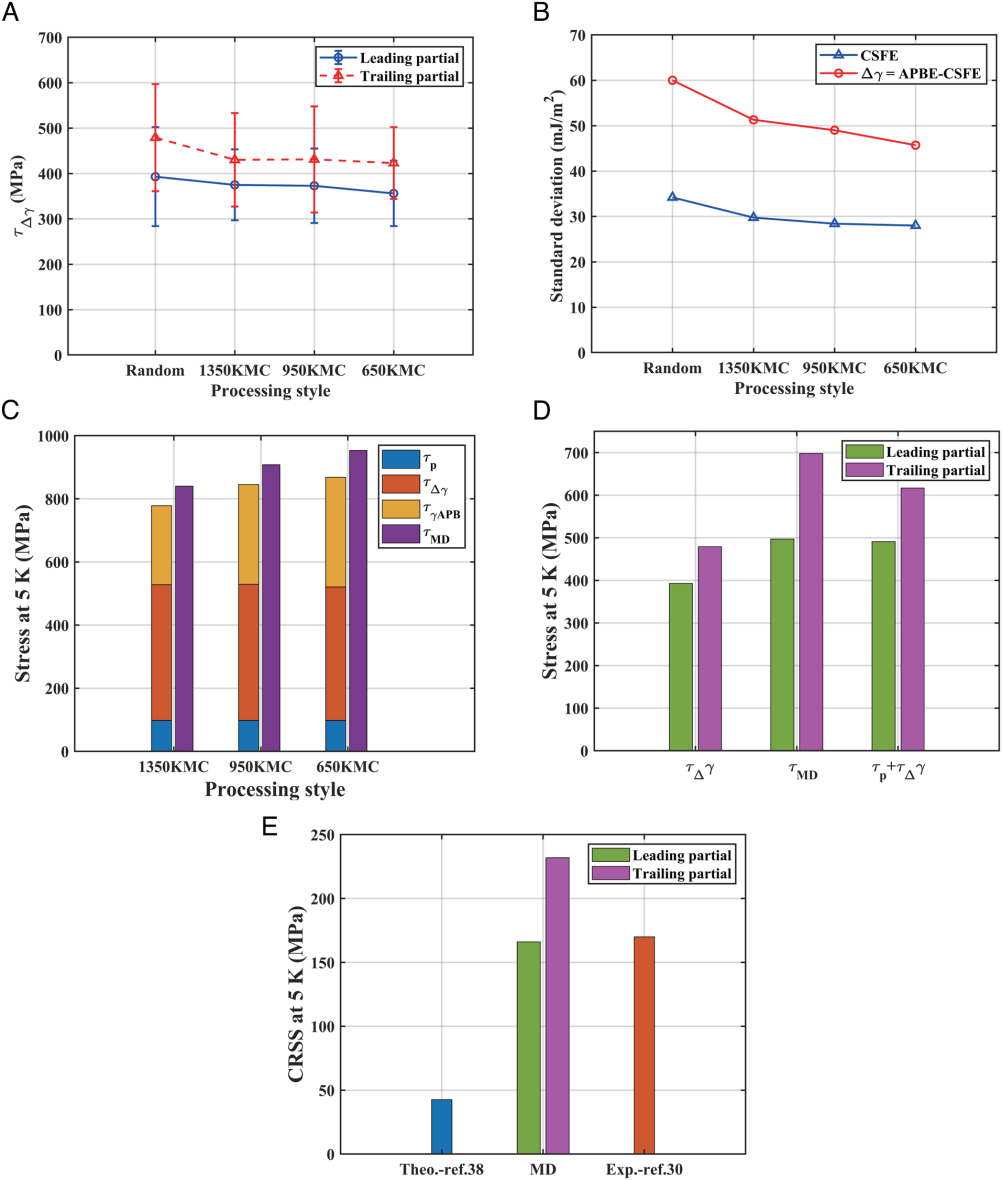
Strengthening due to fault-energy fluctuations. (*A*) Fault-energy fluctuation resistance, $\tau_{\Delta \gamma }$ , for both leading and trailing partials for different SRO states calculated from Eq. **14**. More data given in *SI Appendix*, Fig. S6. (*B*) Fluctuations of CSFE and $\Delta \gamma =\gamma_{\mathrm{APB}}-\gamma_{\mathrm{CSF}}$ on the {111} slip plane. (*C*) Interpretation of MD-predicted CRSS ( $\tau_{MD}$ ) by various strengthening contributions for the different annealed alloy states: $\tau_{p}$ = Peierls stress predicted from Eq. **16**$,\;\tau_{\Delta \gamma }$ = strengthening due to fault-energy fluctuations predicted from Eq. **14**, and $\tau_{\gamma APB}=\gamma_{\mathrm{APB}}/\left(2b\right)$ , the SRO contribution predicted from Eq. **5**. (*D*) Comparison of theoretical predictions with MD results for the random alloy state. Note that for the trailing partial, $\left(\tau_{p}+\tau_{\Delta \gamma }\right)$ includes the small value of $\tau_{\gamma APB}=40$ MPa due to the small $\gamma_{\mathrm{APB}}$ of 11.5 mJ/m^2^ for the random state. (*E*) Comparison of CRSSs obtained from MD simulations for the random alloy state with reported experiments.

The large gap between the MD-predicted CRSS and the Peierls stress can be filled by the strengthening $\tau_{\Delta \gamma }$ arising from the fault-energy fluctuations as proposed in Eq. **14**. To obtain the $\lambda_{i}$ and $\ell_{i}$ in the Fourier series in Eq. **11**, the atomic positions of a wavy partial dislocation were first fitted into a curved line using a smoothing algorithm (28, 29), and then, the computed $w_{i}$ (y), being the difference between two successive dislocation shapes $X_{i}\left(y\right)$ and $X_{i+1}\left(y\right)$ over the segment length *L* as shown in Fig. 3*C*, was fitted to a Fourier series as shown in Fig. 3*E*. As shown in *SI Appendix*, Fig. S6, the calculated fault-energy fluctuation resistance is still a distribution, which arises from the spatial variance of the hard/soft zones on the slip plane. For both the leading and trailing partials, there is remarkable contribution to the CRSS, namely, the $\tau_{\Delta \gamma }$ values are on the order of 300 to 400 MPa, respectively, as shown in Fig. 4*A*. It can be seen that the $\tau_{\Delta \gamma }$ is pretty constant with a slight decreasing trend with increased annealing temperature that tallies with the trend of the fluctuations of the fault energies in Fig. 4*B*. Furthermore, sample calculations of $\tau_{\Delta \gamma }$ were also performed using Eq. **10** without involving the approximations leading to Eq. **14**, but the means and SDs are very similar to those calculated directly using Eq. **14** (c.f. *SI Appendix*, Figs. S6 *A* and *E* and S7 *A* and *B*). These confirm that fluctuations of fault energies play a crucial role in the strength of such CCAs.

Up to this point, as shown in Fig. 4*C*, the MD-predicted CRSS for the annealed alloy states can be interpreted as the summation of the various strengthening contributions discussed above, namely, Peierls stress ( $\tau_{p}$ ) and fault-energy fluctuation resistance ( $\tau_{\Delta \gamma }$ ), together with the SRO strengthening ( $\tau_{\gamma APB}=\gamma_{\mathrm{APB}}/\left(2b\right)$ , from Eq. **5**) which is present for the annealed alloys. However, in terms of magnitude, it can be seen from Fig. 4*C* that the most significant contribution is $\tau_{\Delta \gamma }$ , which makes up 49 to 55% of the total CRSS. Moreover, as mentioned above and illustrated in Fig. 1 *D* and *F*, the SRO strengthening ( $\tau_{\gamma APB}$ ) which appears to be significant is only important for the first dislocation pass and will subdue on further dislocation passes as in macroscopic slip. Therefore, even for an annealed alloy state with initially large SRO strengthening, the real strength in macroscopic deformation is still dominated by the $\tau_{\Delta \gamma }$ contribution which remains on successive dislocation passes (Fig. 1*F*).

The various strengthening contributions are also compared with experiments to understand their importance. The reported experimental CRSS of a NiCoCr single crystal at 14 K is about 170 MPa (30). Considering that the samples in the experiments conducted by Li et al. (30) were annealed at 1,473 K and were close to the random solid-solution state, we took the random state in the MD simulations for comparison. Combining the mean $\tau_{\Delta \gamma }$ values of the random state (393 MPa and 479 MPa for the leading and trailing partials, respectively) with the Peierls stress ( $\tau_{p}=$ 98 MPa), the CRSSs are 491 MPa and 617 MPa (including $\tau_{\gamma APB}=40$ MPa due to the small $\gamma_{\mathrm{APB}}$ of 11.5 mJm^−2^ for the random state) for the leading and trailing partials, respectively, which are in good agreement with that obtained from the MD simulations as shown in Fig. 4*D*. A caveat needs to be noted when comparing the present MD-simulated CRSS to the experimental CRSS. The MD CRSS is calculated for a single dislocation in the simulation cell, but an experimental CRSS measured from a macroscopic sample is for a slip system comprising many dislocations. In the latter case, the group interactions of dislocations will cause a portion of the dislocations to start moving at an applied stress smaller than the Peierls stress of the individual dislocations, and then, this will bring in stress concentrations causing dislocation avalanches and sudden strain bursts (31–34). This effect is thought to explain why MD-calculated CRSSs at 0 K are about three times larger than the experimental values (35, 36), which is also confirmed by discrete dislocation dynamics simulations (37) with a large range of initial dislocation densities, i.e., 10^12^ to 10^16^ $m^{-2},$ that are close to general experimental conditions. In the light of this effect, we apply a correction factor of 1/3 to the MD-simulated CRSSs for single dislocations; the results are 166 MPa for the leading partial dislocation and 232 MPa for the trailing partial dislocation, which are well in line with the experimental CRSS of NiCoCr single crystal at 14 K, i.e., ~170 MPa (30), as shown in Fig. 4*E*. Since $\tau_{p}$ is small, either the $\tau_{\Delta \gamma }$ alone or the superimposed $\left(\tau_{p}+\tau_{\Delta \gamma }\right)$ compares rather favorably with the MD results in Fig. 4*D*, from which the CRSS obtained by applying the 1/3 scaling factor is in accordance with the experimental results as shown in Fig. 4*E*, thus confirming the importance of the $\tau_{\Delta \gamma }$ strengthening.

We note in passing that a solid-solution model (11, 38, 39) also exists in the literature that serves to predict strength in HEAs by taking into account dislocation line-tension effects and size mismatch as follows:

[17] $$\tau_{\mathrm{misfit}}\left(5k\right)=0.0468{\left(\eta G\right)}^{-\frac{1}{3}}{\left(G^{V}\delta \frac{1+\nu }{1-\nu }\right)}^{\frac{4}{3}},$$

where the CRSS at 5 K was approximated as that at 0 K, η = 0.125, δ = 0.02063, and ν=0.268 (38) and $G^{V}=$ 107 GPa is the Voigt shear modulus as listed in *SI Appendix*, Table S1. The calculated macroscopic critical stress for a slip system through the above 1/3 scaling correction is 42.6 MPa as shown in Fig. 4*E*, which is much smaller than the corresponding experimental results.

## 4. Discussion and Conclusion

The simulations and analysis presented above have identified a dominating strength contribution due to fault-energy fluctuations using an EAM interatomic potential for the NiCoCr CCA. Previous understanding of the interactions between dislocations and pinning sites in CCAs was based primarily on solid-solution strengthening models such as that in Eq. **17**, which is inadequate for the present studied alloy as indicated by the comparison in Fig. 4*E*. This probably is because such classical models involve a number of hard-to-justify assumptions, such as equal energy of dislocation segments (hence neglecting irregular positioning of pinning sites on the global slip plane) and neglecting chemical interactions between solutes and dislocation cores. Unlike such models which give a local, characteristic unpinning energy barrier and stress (based primarily on a representative pinned segment and bow-out amplitude), the approach in this work does not rely on any strong assumptions on the energy and spatial positioning of the pinning sites; instead, it simply uses the line tension description of dislocations in conjunction with very general dislocation shapes representable by the Fourier series to fit to MD results to obtain the dislocation resistance (c.f. Eq. **14**). In the present approach, all elastic and chemical nature of the interactions is embodied in the MD simulation data for the dislocation shapes and does not appear as input parameters as in the classical solid-solution strengthening models. The result is a global resistance $\tau_{\Delta \gamma }$ based on a statistical ensemble of dislocation waviness in terms of $\ell_{i}$ and $\lambda_{i}$ obtained directly from MD simulations without any strong assumptions.

The waviness of dislocations was experimentally observed by weak-beam transmission electron microscopy in CCAs including the NiCoCrFeMn alloy^17^ (FCC) and Ti_38_V_15_Nb_23_Hf_24_ alloy (BCC) (40). An important question to answer, at this juncture, is whether the strength contribution $\tau_{\Delta \gamma }$ is unique to the interatomic potential used in the present study. To address this, we have also employed other potentials to simulate the same NiCoCr ternary alloy and the quinary NiCoCrFeMn alloy. For NiCoCr, we have also used a recently developed machine-learning potential (41) to calculate the SFE distribution for the random alloy state, and as shown in *SI Appendix*, Fig. S8, the SFE distribution is similar to that obtained from the present EAM potential, i.e., there is a distribution varying from −170 to 155 mJm^−2^ (c.f. −120 to 100 mJm^−2^ from the EAM potential) with mean SFE −32 mJm^−2^ (c.f. −16 mJm^−2^ from the EAM potential). However, the machine-learning potential is very costly to use—its use has only been demonstrated for systems of several thousand atoms (41, 42) which are too small for dislocation simulations. In addition, we also employed an effective pair potential developed by Groger et al. (43) to perform similar dislocation mobility simulations for the random NiCoCr alloy. The predicted CRSS at 5 K after correction by multiplying the factor of 1/3 is around 90 MPa which is much lower than the experimental values given in Fig. 4*E*, and as shown in *SI Appendix*, Fig. S9*A*, the pair potential predicts a positive mean value for the SFE with very small SD, and the dislocations are very straight during slip. The results suggest that the pair potential is not satisfactory for the NiCoCr alloy, probably due to the fact that it does not account for multiatomic interactions which are important to capture the fluctuation of SFE as confirmed by multiple DFT reports (8, 44, 45). Without the SFE fluctuation properly captured, the strengthening contribution from SFE fluctuations or wavy dislocations would be artificially suppressed, which may explain for the significant underprediction of the CRSS by the pair potential. Employing a similar pair potential to the quinary alloy NiCoCrFeMn (19, 46), the predicted CRSS after the similar correction above is 103 MPa (the critical stress for a single dislocation is about 310 MPa as shown in *SI Appendix*, Fig. S9*B*), which is lower than the experimental single-crystal CRSS at 0 K, i.e., 168 MPa as reported by Kawanura et al. (47). As shown in *SI Appendix*, Fig. S9*B*, the SFE fluctuation is rather weak (at ~ 8 mJm^−2^) compared with that in the NiCoCr alloy (at ~35 mJm^−2^ as given in Fig. 4*B*), with also much less wavy dislocation shapes as simulated by the pair potential that takes no consideration of multiatomic interactions. Therefore, to round up, the SFE fluctuations calculated from the EAM potential for the NiCoCr alloy are consistently reproduced by another, machine-learning, potential (*SI Appendix*, Fig. S8). However, the choice of the potential does affect the prediction—as in the case of ternary NiCoCr, the use of a pair potential would significantly underpredict the CRSS as it artificially suppresses the SFE fluctuation and dislocation waviness (*SI Appendix*, Fig. S9*A*). In a more general context, while the strength contributor of $\tau_{\Delta \gamma }$ is predicted by the present EAM potential to be dominating in the ternary NiCoCr, its significance in other alloys and any governing rule behind will be interesting topics to investigate in the future. In particular, how this strength contributor depends on the alloy complexity and chemistry can be investigated as a data-mining exercise after reliable interatomic potentials have become available for a wider range of CCAs.

Recently, Ostesky and Morris (48) have shown that partial dislocation cores in NiFe alloy tend to favor certain chemical environments, and this effect may manifest itself as local obstacles against dislocation glide. In the present simulations, there was no atom swapping during motion of the dislocations thus, the composition of dislocation cores depends only on the instantaneous location of the dislocation, and the resistance arising from the HAMs depends completely on the spatial distribution of the SFE. From both Eqs. **1** and **2**, one can see that the pinning sites or HAMs correspond to regions of high fault energies, which is confirmed through the local fault-energy statistics as shown in Fig. 2 *G* and *H*. However, atomic exchange via short-circuit diffusion near the dislocation cores may add to the SFE fluctuations, and further work may investigate this type of dynamic obstacles using additional Monte Carlo atom swapping during dislocation motion.

The motivation of the present work is to study single phases of solid solutions with different degrees of SRO, and for this reason, the NiCoCr alloy was annealed at different temperatures to obtain different SRO states. The strongest SRO state was obtained from annealing at 650K, and after MC exchange, the obtained phase was still a single phase. At this low annealing temperature, it is possible that the truly equilibrium alloy state is two phase, although further increasing the MC steps to 8.87 million did not result in significant changes of the potential energy, alloy appearance, and APBE, as shown in *SI Appendix*, Fig. S10 *A* and *B*, and in agreement with previous simulations using the same potential (2), Co-Cr ordering and Ni-Ni clustering are obvious. Furthermore, the computed APBEs after five passes of the <110>a/2 unit dislocation do not exhibit a dramatic jump as reported in ref. 2 (from −50 to 50 mJm^−2^), indicating the stability of the alloy state. The truly equilibrium state of a strongly ordered alloy may be difficult to achieve using MC/MD simulations from small systems, and it is likely that the present alloy state at 650 K is metastable rather than truly stable. In general, if ordering is strong enough to result in a two-phase microstructure, resistance to dislocation motion will be more complicated, and this aspect deserves further investigation.

In conclusion, in the present work, we conducted MD simulations and deduced a model to unfold the physical basis of strength in the metastable NiCoCr alloy at 5 K. The Shockley partials glide in a characteristic jumpy and wavy manner due to successive pinning at sites with high local shear-fault energies associated with special, hard atomic motifs (HAMs) of predominantly Co-Co-Cr-Cr, Co-Co-Co-Cr, or Ni-Cr-Co-Co tetrahedrons. For different SRO states of the alloy achieved by different annealing temperatures, there is an initial strengthening due to the initial fault energy (i.e., CSFE and APBE), which increases with the SRO. However, the APBE and the associated strengthening subdue quickly after multiple passages of dislocations and so are unimportant in macroscopic slip. The basic CRSS which is significant in the random solid-solution state is dominated by the dislocation resistance due to the fluctuations in the local shear-fault energy, which will always remain in the alloy. This strengthening mechanism is unique in CCAs as it is caused by the local fluctuations in the shear-fault energy, corresponding to the HAMs mentioned above, that result in wavy shapes of dislocations of high line energies. The strengthening provided by this mechanism is nearly the entirety of the random alloy strength as the Peierls stress contribution due to lattice misfit distortions is very small. The present identification of the local HAMs as locations of high local shear-fault extends the understanding of dislocation–lattice interactions in CCAs far beyond the traditional concept of the globally averaged SRO parameters. The findings from the present study highlight the effect of fault-energy fluctuations on dislocation waviness and confirm that the fault-energy resistance to dislocation slip is an intrinsic feature of CCAs. The present study not only reveals the unique strengthening mechanism in CCAs but also provides important clues for designing high strength CCAs through tuning the alloy composition to produce large fault-energy fluctuations.

## 5. Materials and Methods

The MD simulations were performed using the Large-scale Atomic/Molecular Massively Parallel Simulator (49) within the three-element Ni-Co-Cr interatomic potential developed by Li et al. (2). Local lattice and defects were identified through common neighbor analysis (CNA) (50) and the centrosymmetric parameter, respectively. The Open Visualization Tool (OVITO) was adopted to visualize dislocation structures (51).

### 5.1. Annealed Simulation Cells.

Hybrid Monte Carlo/Molecular Dynamics (MC/MD) was employed to obtain different alloy species so as to mimic experimental annealing processing as follows. First, considering the computational cost, 2.01 million atoms with equal concentrations of the NiCoCr stoichiometry were randomly distributed in rectangular simulation cells measuring $240\sqrt{2}a$ , $140\sqrt{3/2}a$ , and $5\sqrt{3}a$ (where *a* is the lattice constant of 3.557 Å) along X-$\left[1,\bar{1},0\right]$ , Y-$\left[112\right]$ , and Z-$\left[\bar{1},\bar{1},1\right]$ respectively. Periodic boundary conditions (PBCs) were employed along all three orthogonal directions of X, Y and Z. The simulation cells were then heated up to annealing temperatures of 650, 950, and 1,350 K using the Verlet algorithm and then relaxed under the desired temperatures with the NPT (the number of particles N and pressure P and temperature T are constants) ensemble for 50 ps to reach thermal equilibrium (14). After that, the Monte Carlo (MC) scheme was used to swap atoms inside the cells as per an acceptance criterion of energy reduction under the NPT ensemble at the desired temperatures. To do this, the MC swapping was attempted up to 100 times for every 50 MD steps with a time step of 2.5 fs under the NPT ensemble (4). As shown in *SI Appendix*, Fig. S1 *A* and *B*, both the acceptance rate of MC swapping and potential energy of the annealing samples achieve complete convergence after 6 million MC steps. The calculated Warren–Cowley parameters characterizing the degree of SRO (23) for different annealing temperatures are given in Fig. 1*A*, which are comparable to previous results (2).

### 5.2. Simulations of Dislocation Slip.

The equilibrated configurations after exhaustive MC/MD swapping were then replicated nine times along the Z-1,1,1 direction to generate a series of 30-nm-long simulation cells each comprising 20.3 million atoms as shown in Fig. 1*B* for use in the subsequent simulations for the CRSS of the ½<110> edge dislocation in the NiCoCr alloy. The edge dislocation was introduced parallel to the Y-$\left[112\right]$ direction as shown in Fig. 1*B* according to its anisotropic elastic displacement field, and the simulation cells were subsequently relaxed via a conjugate-gradient scheme to obtain equilibrated dislocation structures comprising two 1/6<112> Shockley partials separated by a stacking fault (14). From this point onward, the hitherto PBCs applied on the Z surfaces (for MC annealing) were changed to free boundary conditions for stress application, while the PBCs were maintained in the other two directions X and Y. To minimize the effects of thermal fluctuations on the dislocation glide and mimic the release of elastic energy during dislocation motion (52), the cells were heated up to 5 K in a quasi-static manner with the NVT ensemble for 50 ps. In order to determine the CRSS, external constant forces were gradually imposed on two thin slabs of thickness 1.2 nm at the lower and upper Z surfaces to produce a desired shear stress $\tau_{\mathrm{xz}}$ through an incremental loading approach (53). Initially, a larger stress increment of 100 MPa was used to quickly identify a rough CRSS, and then, a smaller stress increment of 20 MPa was applied to figure out the more exact CRSS. To minimize shock wave effects, the whole simulation cell was linearly strained under the applied stress, and then, the strained cell was relaxed except for the atoms in two surface thin slabs being fixed. After that, the corresponding external stress was directly applied on the lower and upper thin slabs in which atoms were able to move to respond to the applied stress, and at the same time, relaxation was carried out using an NVT thermostat at 5 K for 200 ps (14, 15).

As indicated in Fig. 1*B*, the length of the simulation cell in the Y direction (the dislocation line direction) was about 600 Å. To ensure that this is sufficiently long to not affect the calculated CRSS (54), convergence tests with shorter lengths of ~400 and ~500 Å were carried out, and it was found that the CRSS results were very similar. The simulations presented in this work were therefore based on the length of about 600Å (~ 240*b*) along the Y direction.

## Supplementary Material

Appendix 01 (PDF)

## Data Availability

All study data are included in the article and/or *SI Appendix*.
