# Supplementary material for: Fluctuations in local shear-fault energy produce unique and dominating strengthening in metastable complex concentrated alloys
Source: Proc Natl Acad Sci U S A. 2023 Mar 13;120(12):e2209188120. doi: 10.1073/pnas.2209188120 (PMC10041143; doi:10.1073/pnas.2209188120)
Supplement: Supplementary file 1 — Appendix 01 (PDF) [file pnas.2209188120.sapp.pdf]

PNAS

Supporting Information for

Fluctuations in local shear-fault energy produce unique and dominating strengthening in metastable complex concentrated alloys

Wei Li, Shuang Lyu, Yue Chen, Alfonso H.W. Ngan

Corresponding author: Alfonso H.W. Ngan

Email: hwngan@hku.hk

This PDF file includes:

Supporting text
Figures S1 to S10
Tables S1
SI References

Supporting Information Text

The calculated elastic constants. The calculated elastic constants for the different annealed samples obtained from MD simulations with different interatomic potentials are summarized in Table S1, which are also compared with experiments and DFT calculations carried out as detailed before[1]. The present studied alloy is rather anisotropic elastically, exhibiting much-larger-one anisotropy ratios of $A = 2C_{44}/(C_{11} - C_{12})$. The shear moduli in the $\langle 110 \rangle$ direction of $\{111\}$ plane, $G_{\{111\}} = [(c_{44}(c_{11} - c_{12})/2]^{1/2}$, are also listed in Table S1. Since the alloy has high elastic anisotropy, the Voigt and Reuss averaged shear moduli G_V and G_R, which are the upper and lower shear modulus bounds, respectively, for polycrystals[2], are also given in Table S1. The elastic constants obtained from MD simulations with the potential proposed by Li et al. (Ref. 2 in the main text) agree well with both DFT and experimental results. In addition, the elastic properties of the annealed samples are larger than that of the random solid-solution state.

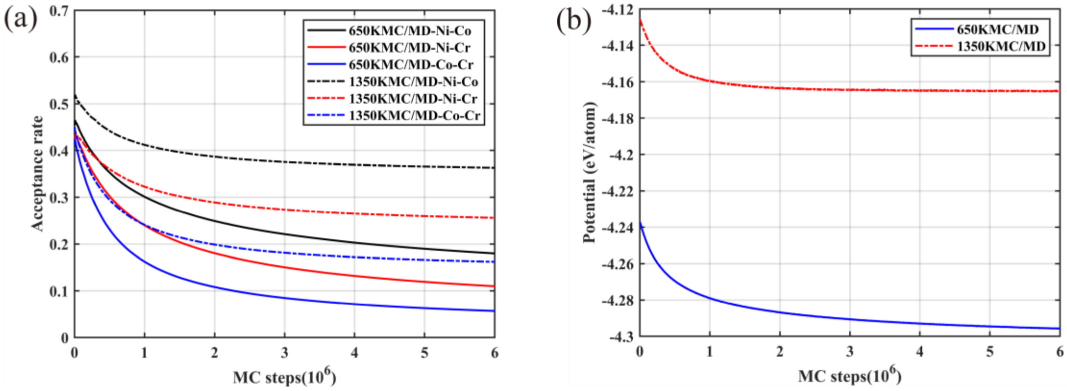

Fig. S1 (a) Acceptance rate of MC swapping and (b) potential energy per atom vs MC steps.

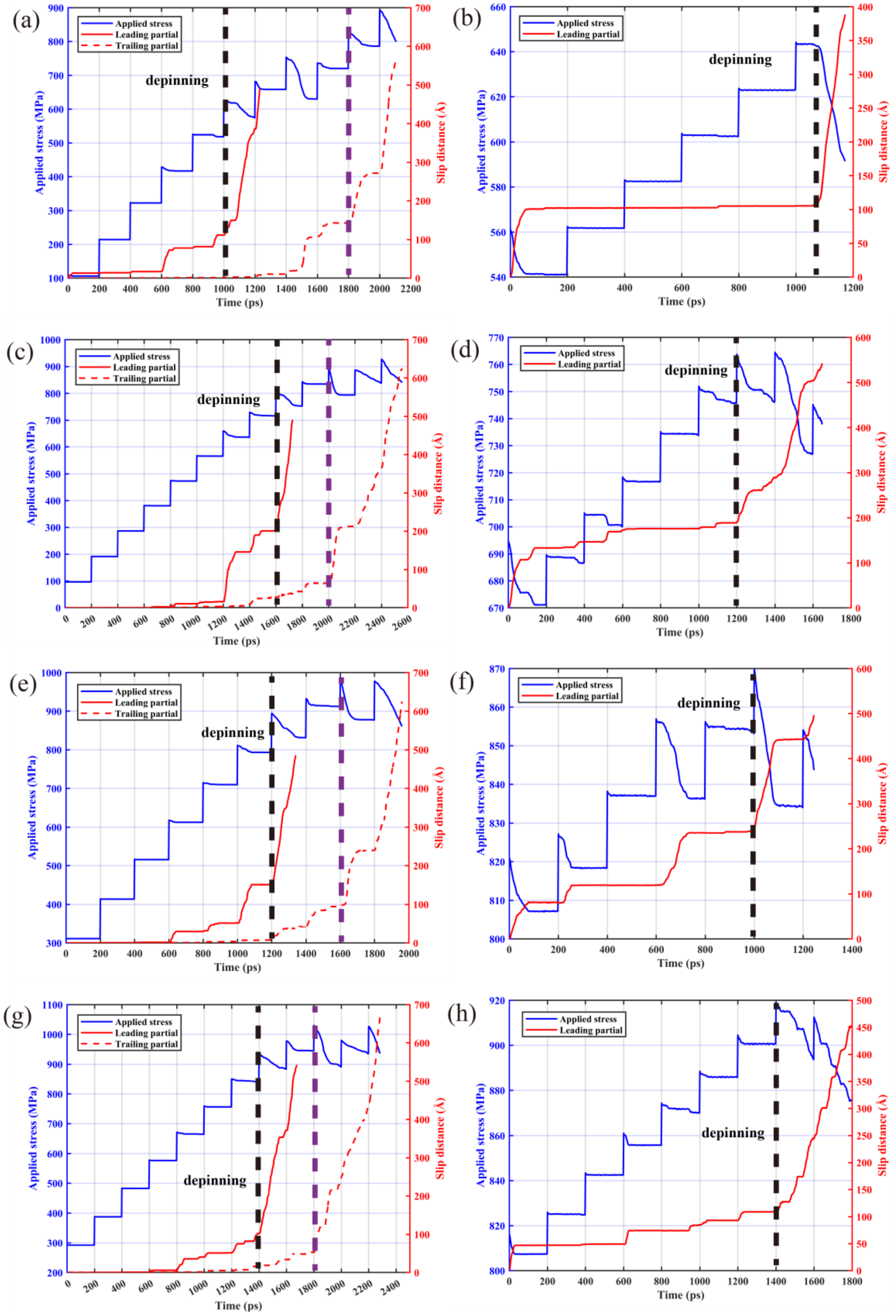
(a)
(b)
(c)
(d)
(e)
(f)
(g)
(h)
Applied stress
Leading partial
Trailing partial
depinning
Applied stress (MPa)
Slip distance (Å)
Time (ps)

Fig. S2 Applied stress and average positions of the leading and trailing partials obtained from MD simulations at 5 K. (a), (c), (e) and (g) correspond the random state, 1350, 950 and 650 K annealed states respectively for stress increments of 100MPa, (b),(d),(f) and (h) correspond the random state, 1350, 950 and 650 K annealed states respectively for stress increments of 20MPa. Dashed lines denote depinning states.

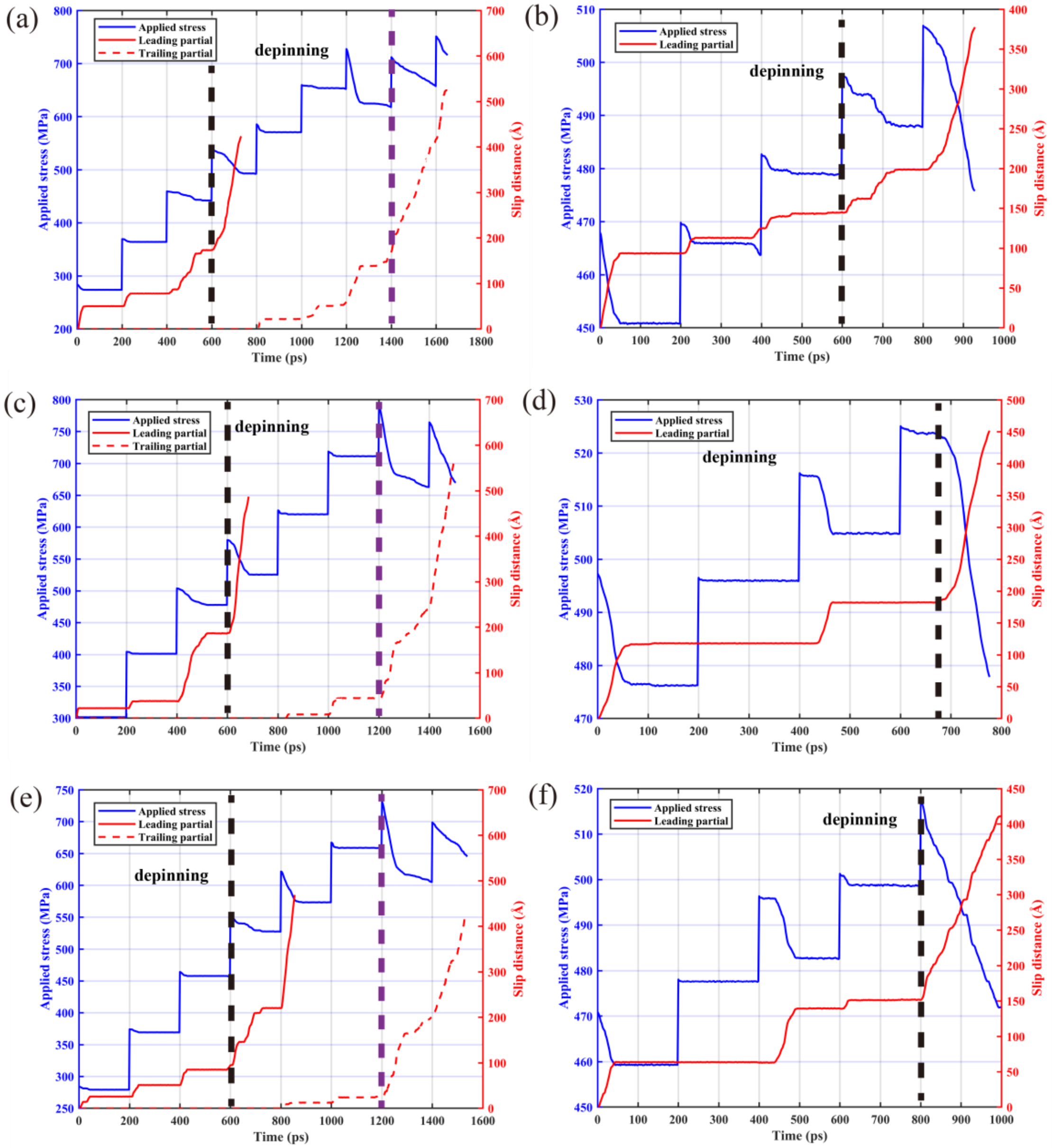

Fig. S3 Applied stress and average positions of the leading and trailing partials after multiple passages (5 times) of dislocations obtained from MD simulations at 5 K. (a), (c) and (e) correspond 650, 950 and 1350 K annealed states respectively for stress increments of 100MPa, (b), (d) and (f) correspond 650, 950 and 1350 K annealed states respectively for stress increments of 20MPa. Dashed lines denote depinning states.

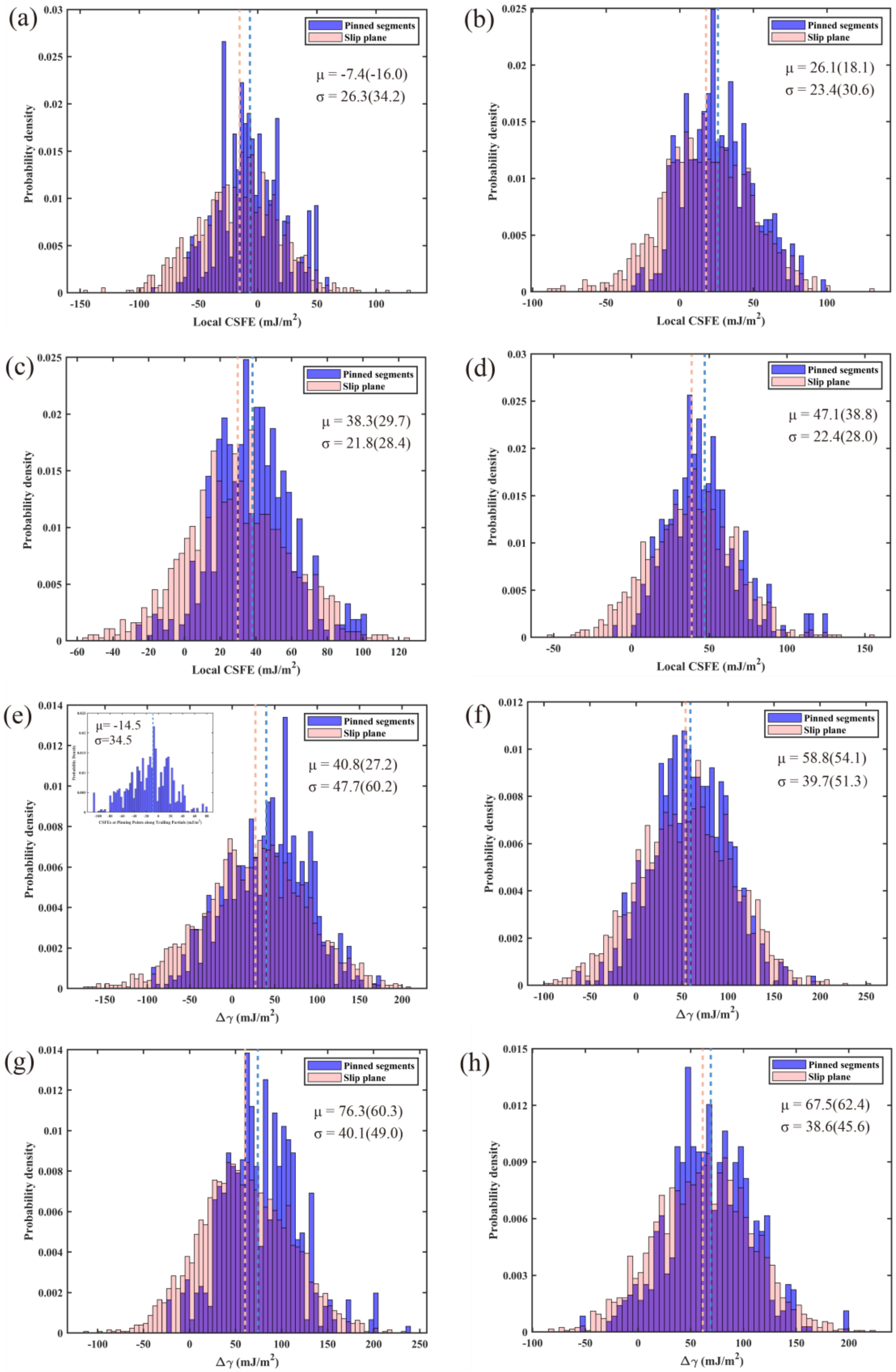
(a)
Pinned segments
Slip plane
μ = -7.4(-16.0)
σ = 26.3(34.2)
Probability density
Local CSFE (mJ/m²)
(b)
Pinned segments
Slip plane
μ = 26.1(18.1)
σ = 23.4(30.6)
Probability density
Local CSFE (mJ/m²)
(c)
Pinned segments
Slip plane
μ = 38.3(29.7)
σ = 21.8(28.4)
Probability density
Local CSFE (mJ/m²)
(d)
Pinned segments
Slip plane
μ = 47.1(38.8)
σ = 22.4(28.0)
Probability density
Local CSFE (mJ/m²)
(e)
μ= -14.5
σ=34.5
Pinned segments
Slip plane
μ = 40.8(27.2)
σ = 47.7(60.2)
Probability density
Δγ (mJ/m²)
(f)
Pinned segments
Slip plane
μ = 58.8(54.1)
σ = 39.7(51.3)
Probability density
Δγ (mJ/m²)
(g)
Pinned segments
Slip plane
μ = 76.3(60.3)
σ = 40.1(49.0)
Probability density
Δγ (mJ/m²)
(h)
Pinned segments
Slip plane
μ = 67.5(62.4)
σ = 38.6(45.6)
Probability density
Δγ (mJ/m²)

Fig. S4 Local CSFEs, (a)-(d) for random, 1350, 950 and 650 K annealed samples respectively, and energy differences between CSFEs and APBEs ($\Delta\gamma$), (e)-(h) for random, 1350, 950 and 650 K annealed samples respectively, around the pinned dislocation segments for the leading and trailing partials respectively. (μ and σ are mean and standard deviation in mJm^{-2}, respectively; values in parentheses are for the whole slip plane. Dash lines denote the mean values).

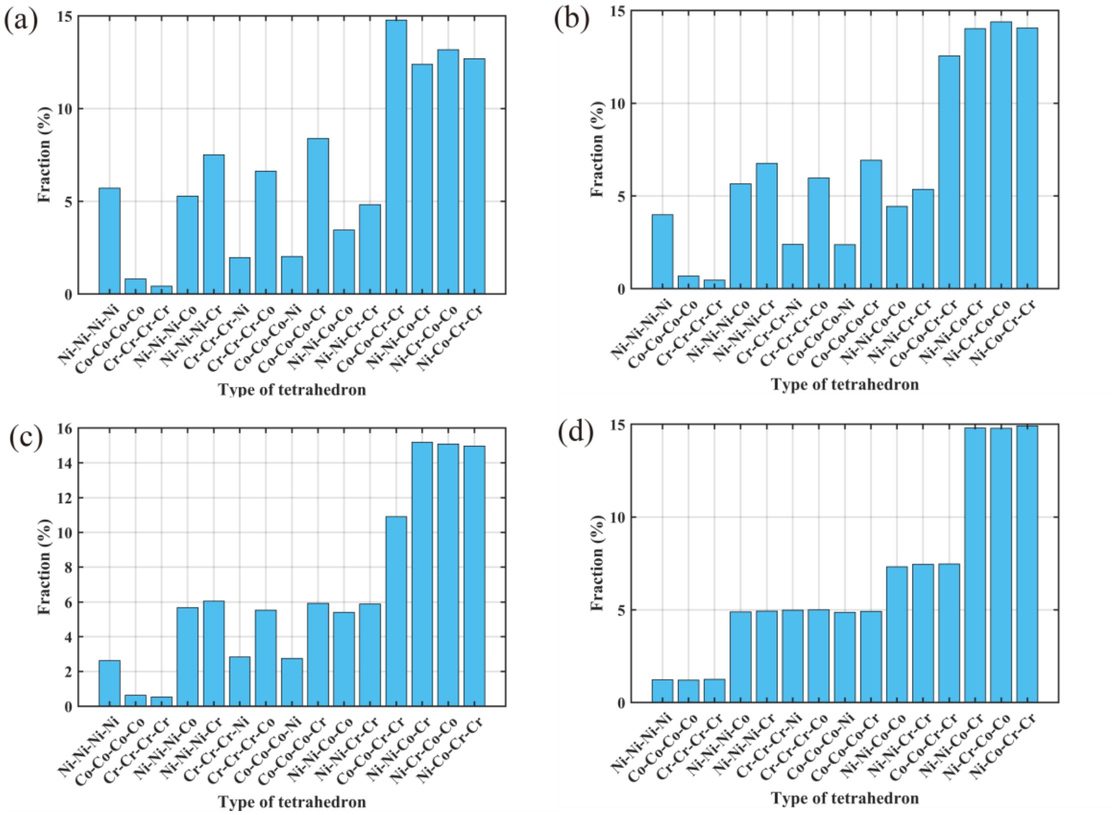

Fig. S5 Tetrahedron distribution on four parallel {111} planes around the slip plane, (a)-(d) for 650, 950 and 1350 K annealed samples and random sample respectively.

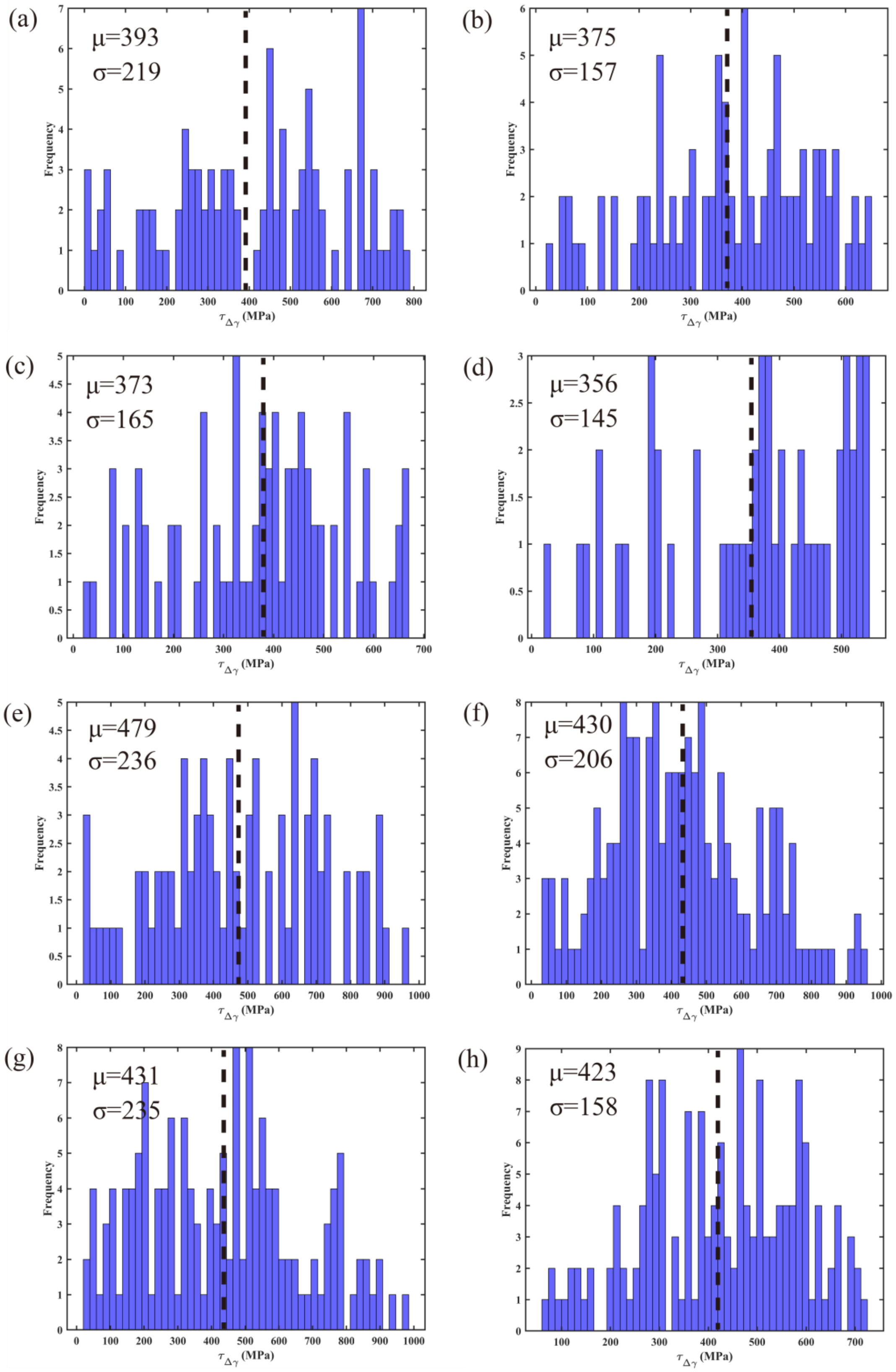

(a)
μ=393
σ=219
(b)
μ=375
σ=157
(c)
μ=373
σ=165
(d)
μ=356
σ=145
(e)
μ=479
σ=236
(f)
μ=430
σ=206
(g)
μ=431
σ=235
(h)
μ=423
σ=158
Frequency
$\tau_{\Delta\gamma}$ (MPa)

Fig. S6 Strengthening from fault-energy fluctuations $\tau_{\Delta\gamma}$. (a)-(d) for the leading partials in random, 1350, 950 and 650 K annealed samples respectively, and (e)-(h) for the trailing partials in random, 1350, 950 and 650 K annealed samples respectively. (μ and σ denote mean and standard deviation in MPa, respectively).

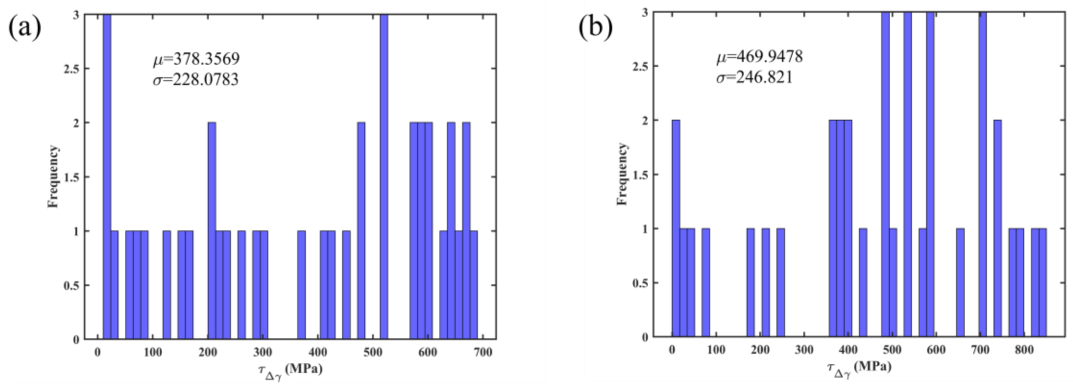

Fig. S7 $\tau_{\Delta\gamma}$ obtained from Eqn. (10) for the alloy in random state, for (a) leading partial dislocations, and (b) trailing partial dislocations.

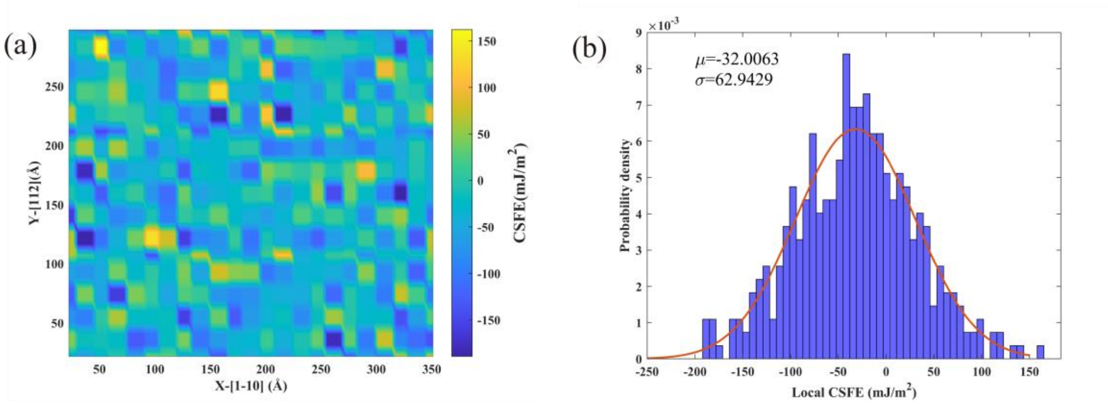

Fig. S8 Local SFE distribution of NiCoCr random alloy calculated using a machine-learning potential (Refs.41 and 42 in the main text).

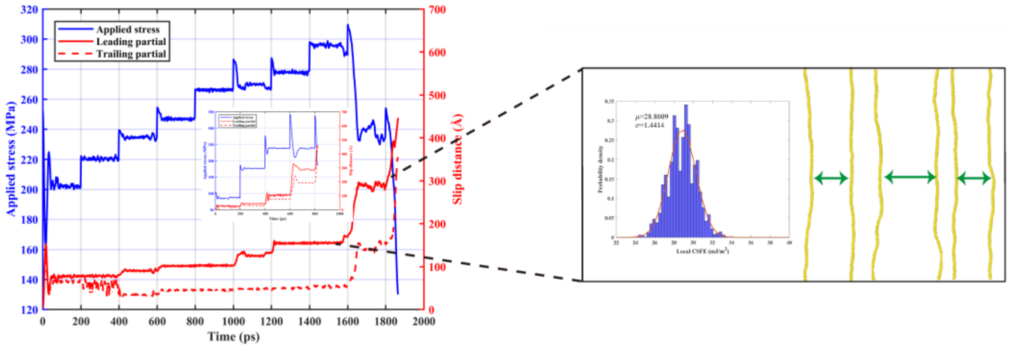

(a)

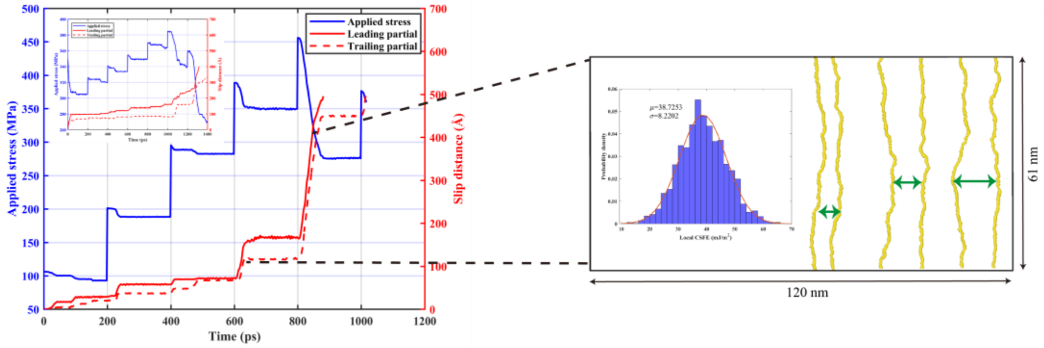

(b)

Fig. S9 Applied stress and average positions of the leading and trailing partials obtained from MD simulations at 5 K for (a) random NiCoCr, and (b) random NiCoCrFeMn, both with the effective pair potential (Ref.43 in the main text).

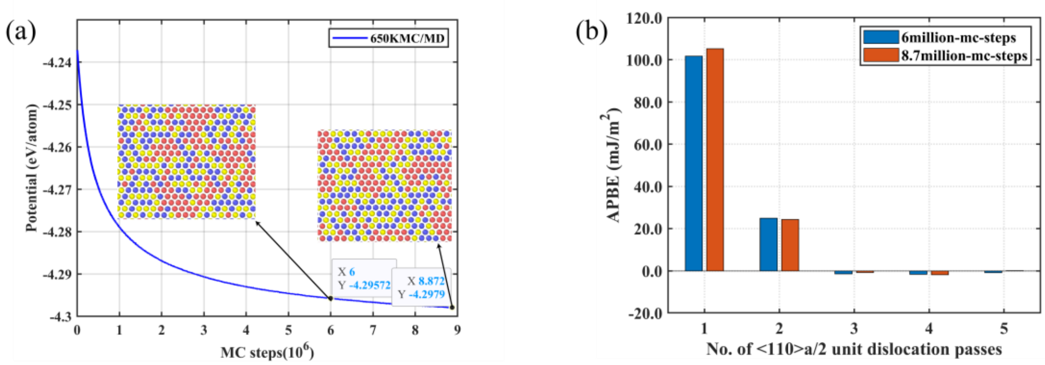

Fig. S10 Effects of MC steps on (a) potential energy and alloy appearance, and (b) APBE, during 650 K annealing of NiCoCr using EAM potential. In (a), Ni atoms are in red, Co atoms are in blue, and Cr atoms are in yellow.

Table S1. Elastic constants (GPa) for different annealed samples as predicted using DFT calculations and MD simulations with different potentials.

Method	C_{11}	C_{12}	C_{44}	$(C_{11}-C_{12})/2$	A	G{111}	G^V	G^R
Exp._300K[3]	249	156	142	47	3.1	81	104	78
Exp._0K[4]	255	159	147	48	3	84	107	81
DFT	262	159	127	52	2.5	81	97	80
MD_Aver._Rand.	197	157	115	20	5.8	48	77	40
MD_Farkas_Rand.[5]	208	147	128	31	4.2	62	89	56
MD_Meam_Rand.[6]	232	156	74	38	1.9	53	60	54
MD_Li_Rand.	251	177	94	37	2.5	59	71	58
MD_Li_1350KMC	268	182	101	43	2.3	66	78	66
MD_Li_950KMC	260	183	101	39	2.6	62	76	61
MD_Li_650KMC	270	185	103	43	2.4	66	79	66

SI References

1. W. Li, X. Peng, A. H. W. Ngan, J. A. El-Awady, Surface energies and relaxation of NiCoCr and NiFeX (X = Cu, Co or Cr) equiatomic multiprincipal element alloys from first principles calculations. Modelling and Simulation in Materials Science and Engineering 30, 025001 (2022).
2. Mehl, M. J., Osburn, J. E., Papaconstantopoulos, D. A. & Klein, B. M. Structural properties of ordered high-melting-temperature intermetallic alloys from first-principles total-energy calculations. Phys. Rev. B Condens. Matter 41, 10311–10323 (1990).
3. Jin, K., Gao, Y. F. & Bei, H. Intrinsic properties and strengthening mechanism of monocrystalline Ni-containing ternary concentrated solid solutions. Materials Science and Engineering: A vol. 695 74–79 (2017).
4. Laplanche, G. et al. Processing of a single-crystalline CrCoNi medium-entropy alloy and evolution of its thermal expansion and elastic stiffness coefficients with temperature. Scripta Materialia vol. 177 44–48 (2020).
5. Farkas, D. & Caro, A. Model interatomic potentials and lattice strain in a high-entropy alloy. Journal of Materials Research vol. 33 3218–3225 (2018).
6. Choi, W.-M., Jo, Y. H., Sohn, S. S., Lee, S. & Lee, B.-J. Understanding the physical metallurgy of the CoCrFeMnNi high-entropy alloy: an atomistic simulation study. npj Computational Materials vol. 4 (2018).
